# Recurrent Idiopathic Intracranial Hypertension in a Patient With Systemic Lupus Erythematosus: A Case Report

**DOI:** 10.1002/ccr3.73287

**Published:** 2026-08-09

**Authors:** Leila Poorsaadat, Baharak Tasorian, Mohammad Sadegh Fakhari, Reza Mohammadi

**Affiliations:** ^1^ Department of Neurology, School of Medicine Arak University of Medical Sciences Arak Iran; ^2^ Department of Rheumatology, School of Medicine Arak University of Medical Sciences Arak Iran; ^3^ Department of Neurology Arak University of Medical Sciences Arak Iran

**Keywords:** case report, idiopathic intracranial hypertension, pseudotumor cerebri, systemic lupus erythematosus

## Abstract

Idiopathic intracranial hypertension (IIH) is a rare but significant complication in patients with systemic lupus erythematosus (SLE). Its diagnosis and management can be challenging, particularly in cases of recurrence. We report the case of a 26‐year‐old Persian female with a history of SLE and hypertension who presented with persistent headaches and bilateral papilledema. Diagnostic workup, including lumbar puncture, revealed elevated cerebrospinal fluid (CSF) pressure consistent with IIH, without evidence of infection or other secondary causes. Initial treatment with acetazolamide led to symptom resolution and normalization of CSF pressure. However, following the discontinuation of acetazolamide after a year of symptom‐free follow‐up, she experienced a recurrence of IIH. High‐dose acetazolamide was readministered, resulting in remission. The patient was managed with continued follow‐up and a multidisciplinary approach, including adjustments to her SLE and hypertension treatments. This case underscores the importance of long‐term monitoring in patients with SLE who develop IIH. It highlights the potential for IIH recurrence after treatment withdrawal, emphasizing the need for individualized management strategies and a multidisciplinary approach to ensure optimal outcomes.

**Key Clinical Message**

This case highlights the need for vigilance in managing idiopathic intracranial hypertension (IIH) in patients with systemic lupus erythematosus (SLE), emphasizing the potential for recurrence upon treatment withdrawal and the importance of regular monitoring and a multidisciplinary approach for optimal outcomes.

## 1 Background

Headache is a common symptom in patients with Systemic Lupus Erythematosus (SLE), arising from various etiologies. Among these, Idiopathic Intracranial Hypertension (IIH) is a rare but notable cause, reported in approximately 1% of patients with SLE [1]. IIH is characterized by symptoms of intracranial hypertension, including positional headache, nausea, vomiting, papilledema, visual disturbances, and sixth cranial nerve palsy. Diagnosis is established based on the Dandy criteria, which require clinical features of intracranial hypertension, brain imaging without significant abnormalities, and normal CSF analysis except for elevated opening pressure [2].

IIH has been linked to factors such as glucocorticoid withdrawal, hypercoagulable states, SLE disease activity, and, in some reports, the presence of anti‐Ro antibodies [1, 3]. Although several cases of IIH associated with SLE have been documented, the pathogenesis remains unclear [4]. Proposed mechanisms include aseptic meningitis, immune complex deposition on the arachnoid villi, and micro‐occlusion of these villi [5]. Recent advances in management—such as targeted immunosuppression and cerebrospinal fluid diversion techniques—have improved prognosis; however, definitive diagnosis and optimal treatment regimens remain challenging [6].

This case report highlights the clinical course, challenges, and outcomes in managing recurrent IIH in a patient with SLE, emphasizing the importance of a multidisciplinary approach in addressing this rare manifestation.

## 2 Case History and Examination

A 26‐year‐old married Persian woman (body mass index, 24 kg/m^2^) with a history of SLE and well‐controlled hypertension was referred to our neurology clinic in September 2021 because of persistent headache. She had been diagnosed with SLE in 2017 based on the 2012 Systemic Lupus International Collaborating Clinics (SLICC) classification criteria, fulfilling three clinical criteria (malar rash, inflammatory arthritis, and myositis) and two immunologic criteria (positive antinuclear antibody [ANA] at a titer of 1:160 on HEp‐2 cells and positive anti‐double‐stranded DNA [anti‐dsDNA] antibodies). Complement levels (C3/C4) had not been measured because the diagnosis was established using the available classification criteria. At presentation, her medications included hydroxychloroquine, azathioprine, prednisolone, losartan, and diltiazem.

On neurological examination, the findings were unremarkable except for bilateral papilledema, which was confirmed by ophthalmologic evaluation. The patient also had an active cutaneous rash, inflammatory arthritis, and myositis, corresponding to a Systemic Lupus Erythematosus Disease Activity Index 2000 (SLEDAI‐2K) score of 10.

## 3 Methods

### 3.1 Differential Diagnosis

The differential diagnosis included cerebral venous sinus thrombosis, intracranial mass lesion, meningitis, neuropsychiatric lupus, medication‐induced intracranial hypertension, and IIH.

### 3.2 Investigations

Laboratory investigations demonstrated mild anemia and an elevated erythrocyte sedimentation rate (36 mm/h), whereas renal function remained normal. Proteinuria exceeding 1000 mg/day was detected on a single occasion; however, repeat urine protein quantification was < 500 mg/day. Urinalysis revealed no active urinary sediment (including hematuria or urinary casts), urine culture was negative, and serum creatinine and blood urea nitrogen remained within the normal range. Because the proteinuria was transient and there was no evidence of persistent renal involvement or active urinary sediment, lupus nephritis was considered unlikely, and renal biopsy was not indicated. Antiphospholipid syndrome screening was negative, while anti‐dsDNA antibodies remained qualitatively positive.

Brain magnetic resonance imaging (MRI) and magnetic resonance venography (MRV) excluded intracranial mass lesions and cerebral venous sinus thrombosis. Lumbar puncture was performed because of suspected intracranial hypertension and demonstrated an opening pressure of 30 cm H_2_O. Cerebrospinal fluid (CSF) analysis was otherwise normal, with no evidence of infection or inflammation. Based on the revised Dandy criteria [7], a diagnosis of IIH was established.

### 3.3 Treatment

The patient was treated with oral acetazolamide, resulting in complete resolution of headache and gradual improvement of papilledema. She continued regular follow‐up in both neurology and rheumatology clinics with serial ophthalmologic examinations, including funduscopy, visual field testing, and optical coherence tomography (OCT). Hydroxychloroquine was discontinued in July 2022 because of ocular toxicity unrelated to intracranial hypertension and was replaced with methotrexate (7.5 mg weekly). Her maintenance treatment also included prednisolone (2.5 mg/day), losartan (50 mg twice daily), diltiazem (60 mg twice daily), spironolactone (25 mg/day), and vitamin D supplementation.

### 3.4 Follow‐Up and Recurrence

Following complete clinical remission, acetazolamide was gradually tapered and discontinued after 2 years of treatment. The patient remained asymptomatic until November 2024, when she presented with recurrent persistent headache and was referred to our neurology clinic.

Neurological examination again demonstrated bilateral papilledema without focal neurological deficits. Compared with the initial presentation, lupus disease activity was lower, with active rash and arthritis corresponding to a SLEDAI‐2K score of 6. The Systemic Lupus International Collaborating Clinics/American College of Rheumatology Damage Index (SDI) had increased to 1 because of persistent visual impairment. Laboratory investigations showed preserved renal function, stable inflammatory markers, and persistently positive qualitative anti‐dsDNA antibodies (Table 1).

**TABLE 1:** Laboratory results during two admissions for headache.

Date	12 September 2021	24 November 2024
Blood sample		
White Blood Cell (×10^3^/μL)	5.6	4.6
Hemoglobin (gr/dL)	11.2	10.5
Platelet (×10^3^/μL)	218	212
Prothrombin time (seconds)	10.5	14
Partial thromboplastin time (seconds)	42	42
Blood urea nitrogen (mg/dL)	14.79	17
Creatinine (mg/dL)	1.1	1.2
Sodium (meq/L)	141	137
Potassium (meq/L)	3.9	3.76
Erythrocyte sedimentation rate (mm)	36	25
C reactive protein	3+	3+
Lumbar puncture		
Glucose (mg/dL)	45	46
Protein	18.5	30
White blood cell (×10^3^/μL)	0	3
Red blood cell (×10^6^/μL)	110	20
Lactate dehydrogenase (Iu/L)	52	27
Culture (after 48 h)	No growth	—
Pressure (cm H_2_O)	30	40
SLE activity parameters		
SLEDAI	10 (rash, arthritis, myositis)	6 (rash, arthritis)
SDI	0	1 (visual impairment)
Anti‐dsDNA antibodies	Positive	Positive

Abbreviations: SDI, Systemic Lupus International Collaborating Clinics/American College of Rheumatology Damage Index; SLEDAI; SLE Disease Activity Index.

**FIGURE 1:**
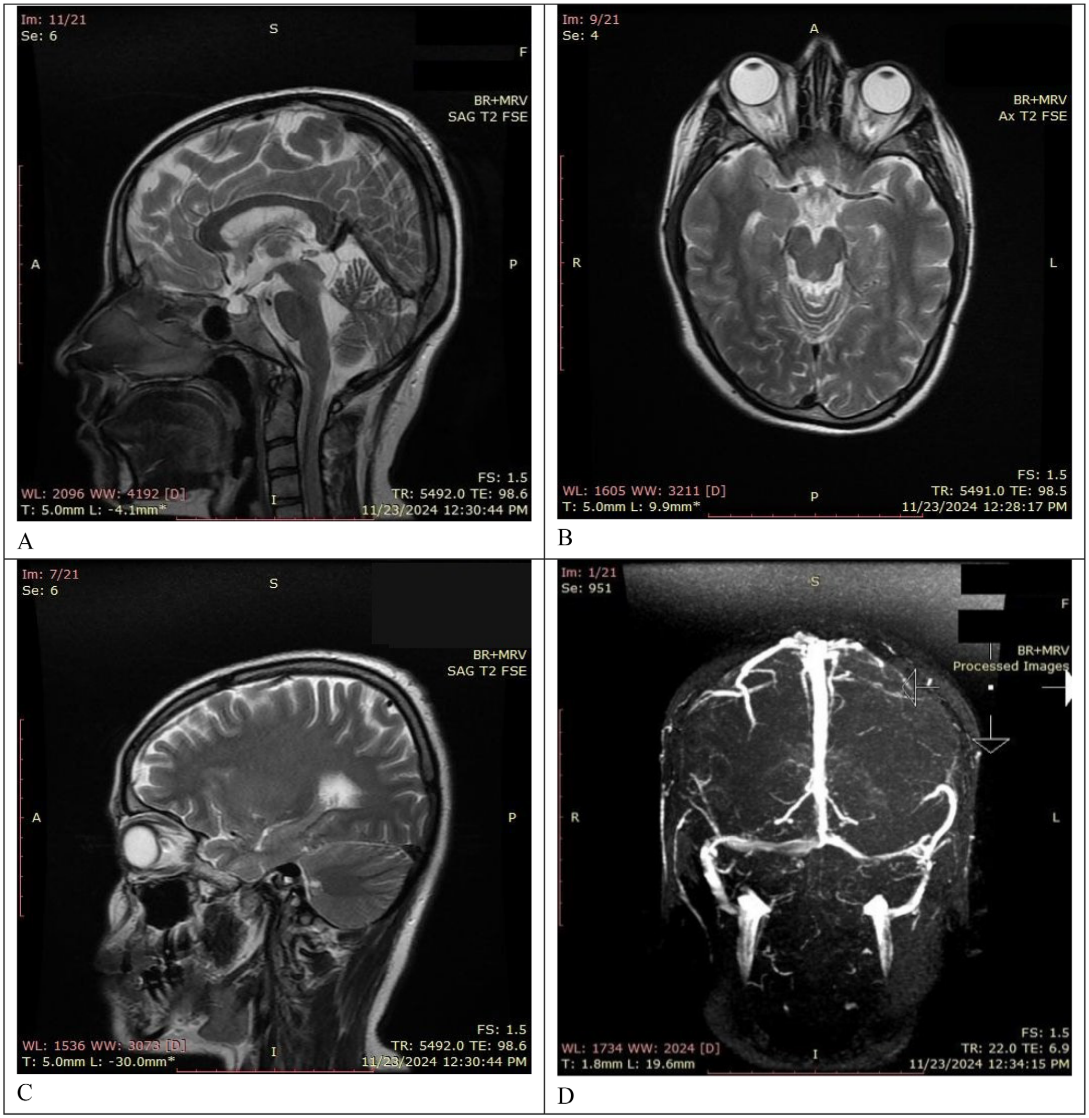
Neuroimaging findings during the second episode of idiopathic intracranial hypertension. (A) Sagittal T2‐weighted MRI demonstrating a partial empty sella with flattening of the pituitary gland. (B) Axial T2‐weighted MRI demonstrating distension of the perioptic cerebrospinal fluid spaces (optic nerve sheath enlargement). (C) Sagittal T2‐weighted MRI demonstrating mild tortuosity of the optic nerve, consistent with elevated intracranial pressure. (D) Coronal MR venography demonstrating focal transverse sinus narrowing/collapse without evidence of cerebral venous sinus thrombosis.

Repeat brain MRI demonstrated a partial empty sella, distension of the perioptic subarachnoid spaces with mild tortuosity of both optic nerves, while MRV demonstrated focal transverse sinus narrowing without evidence of cerebral venous sinus thrombosis (Figure 1). Repeat lumbar puncture revealed an opening pressure of 40 cm H_2_O, whereas CSF composition remained normal, confirming recurrent IIH.

High‐dose acetazolamide was reintroduced, leading to rapid resolution of headache and normalization of intracranial pressure. Immunosuppressive therapy was modified to prednisolone (5 mg/day) and mycophenolate mofetil (500 mg each morning and 1000 mg each evening), while antihypertensive therapy was continued.

**FIGURE 2:**
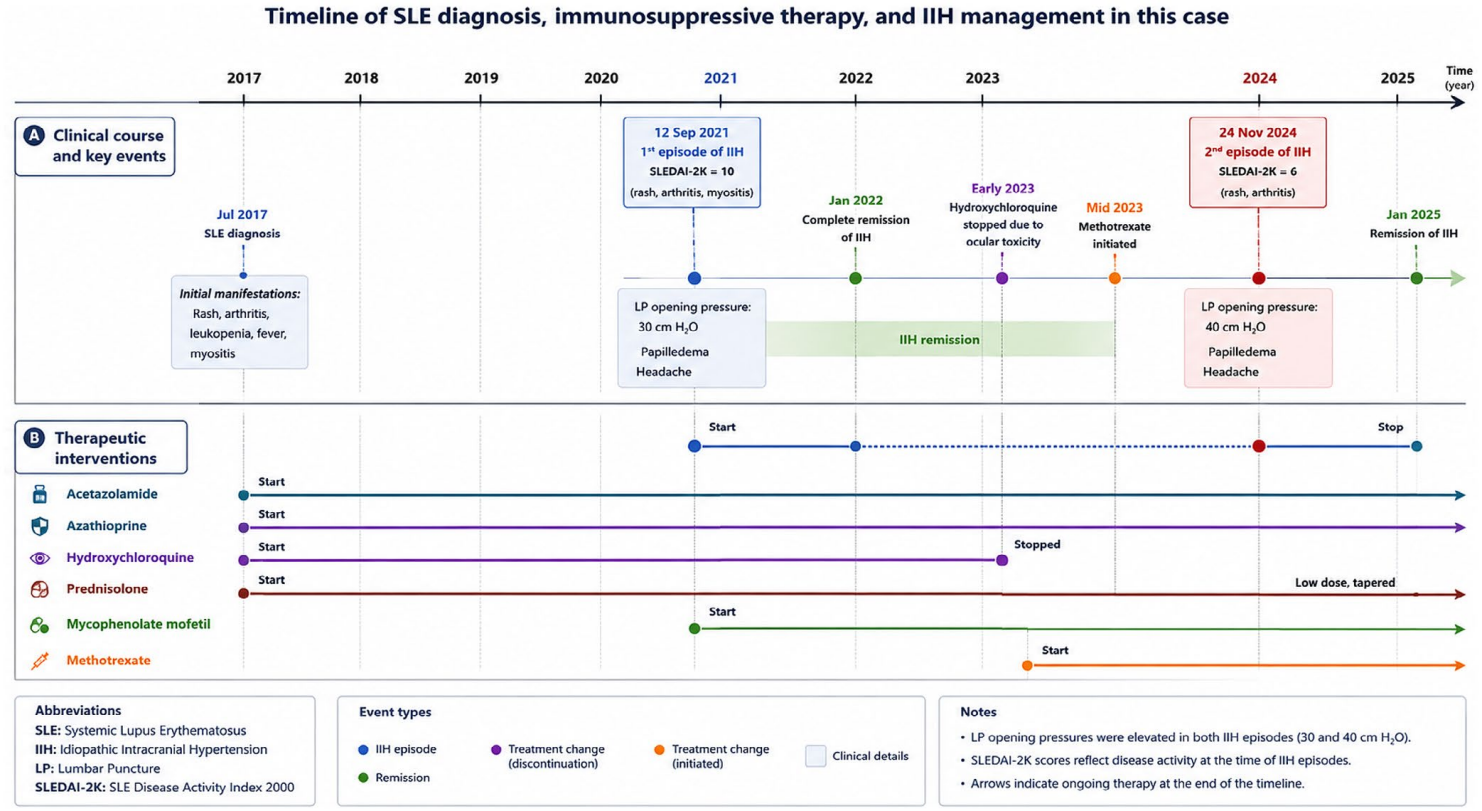
Timeline of SLE diagnosis, immunosuppressive therapy, and IIH management.

At 1‐year follow‐up, the patient remained free of recurrent headache or papilledema. Serial ophthalmologic examinations demonstrated progressive improvement in papilledema, and automated visual field testing showed marked recovery of the previously documented bilateral scotomas. Serial visual field and OCT examinations likewise demonstrated improvement in optic nerve head edema ([Link], [Link]). The chronological relationship between SLE disease activity, IIH episodes, and therapeutic interventions is summarized in Figure 2, and detailed laboratory and disease activity parameters are presented in Table 1.

## 4 Discussion

IIH is an uncommon but well‐recognized neurological manifestation of SLE. Although rare, it may result in disabling headaches, papilledema, and permanent visual impairment if not diagnosed and treated promptly. This case highlights the diagnostic and therapeutic challenges posed by recurrent IIH in a patient with established SLE and emphasizes the importance of considering both disease‐related and treatment‐related mechanisms.

The pathogenesis of IIH in SLE remains incompletely understood and is likely multifactorial. Immune‐mediated inflammation of the arachnoid villi, the primary site of CSF absorption, has been proposed as a mechanism leading to impaired CSF resorption and elevated intracranial pressure [8]. In addition, SLE is associated with a prothrombotic state that may promote microvascular occlusion of cerebral venous channels, thereby increasing resistance to CSF drainage even in the absence of overt cerebral venous sinus thrombosis [8]. Previous studies have identified female sex, active serologic disease, lupus nephritis, previous thrombotic events, and antiphospholipid antibodies as factors associated with an increased risk of IIH in patients with SLE [9].

Drug‐induced intracranial hypertension should also be considered in patients with SLE because several medications have been implicated in its development. Corticosteroid withdrawal is a well‐recognized precipitating factor and represents the strongest pharmacologic association reported in the literature [3]. Isolated case reports have also linked methotrexate and mycophenolate mofetil to intracranial hypertension in patients with autoimmune diseases or following organ transplantation [10, 11, 12, 13]. In contrast, current evidence does not support a causal association between azathioprine or hydroxychloroquine and IIH, as neither drug has been consistently implicated in pharmacovigilance studies or systematic reviews [14, 15]. Consequently, distinguishing disease‐related IIH from medication‐induced intracranial hypertension may be challenging and requires careful evaluation of disease activity, medication history, and the temporal relationship between treatment modifications and symptom onset.

Our patient illustrates this diagnostic complexity. During the initial episode, IIH developed in the setting of more active lupus (SLEDAI‐2K score of 10), suggesting that systemic inflammation may have contributed to the development of intracranial hypertension. By contrast, the recurrent episode occurred despite lower overall lupus activity (SLEDAI‐2K score of 6), making uncontrolled systemic disease a less likely explanation. Furthermore, the only major therapeutic change preceding recurrence was escalation of mycophenolate mofetil approximately 1 month before symptom onset. Although a causal relationship cannot be established from a single case, the temporal association raises the possibility that medication exposure may have contributed to recurrence. Nevertheless, this observation should be interpreted cautiously given the limited evidence linking mycophenolate mofetil to IIH.

The recurrence of IIH in our patient was likely multifactorial, reflecting the interplay between disease‐related susceptibility and potential treatment‐related factors. Recurrent IIH in patients with SLE has been reported only rarely, and previous studies have identified female sex, active serologic disease, and corticosteroid withdrawal as potential predisposing factors [3, 16, 17, 18]. Although our patient demonstrated moderate lupus activity at the time of recurrence (SLEDAI‐2K score of 6), her disease activity was lower than during the initial episode (SLEDAI‐2K score of 10), and there was no evidence of progressive renal involvement or other major systemic manifestations to suggest a lupus flare as the primary driver of recurrence. Furthermore, anti‐dsDNA antibodies remained qualitatively positive without significant clinical deterioration, and the transient proteinuria observed previously had not progressed. Notably, the only major therapeutic change preceding the second IIH episode was escalation of mycophenolate mofetil approximately 1 month before symptom onset. Although isolated cases of mycophenolate‐associated intracranial hypertension have been described in pediatric autoimmune disorders and transplant recipients [12], the available evidence remains limited, and a causal relationship cannot be established from a single observation. Nevertheless, the temporal association in our patient raises the possibility that mycophenolate mofetil may have acted as a contributing factor in a patient already predisposed to IIH. This case therefore highlights the importance of carefully evaluating both lupus disease activity and recent therapeutic modifications when assessing recurrent intracranial hypertension in patients with SLE. Similar diagnostic challenges have been described in other uncommon neurological presentations, underscoring the value of maintaining a broad differential diagnosis [19].

Most reported cases of SLE‐associated IIH respond favorably to medical therapy, particularly when recognized early. Treatment generally consists of controlling both intracranial hypertension and the underlying autoimmune disease. Acetazolamide remains the first‐line agent for reducing CSF production, whereas glucocorticoids may be appropriate in patients with active lupus or suspected inflammatory mechanisms. Surgical procedures, including optic nerve sheath fenestration or cerebrospinal fluid diversion, should be reserved for patients with progressive visual deterioration or medically refractory disease [1, 20].

This case also highlights the importance of long‐term multidisciplinary follow‐up. Serial ophthalmologic assessment, including funduscopic examination, optical coherence tomography, and visual field testing, was essential for documenting disease progression and treatment response. The recurrence of IIH despite previous remission emphasizes that prolonged clinical surveillance is warranted in patients with SLE, particularly following changes in immunosuppressive therapy or corticosteroid tapering.

Although this report describes a single patient and cannot establish causality, it contributes to the limited literature on recurrent IIH in SLE. The availability of serial neuroimaging, lumbar puncture findings, longitudinal ophthalmologic assessments, and lupus disease activity measures strengthens the clinical documentation of this case. Additional studies are needed to clarify the relative contributions of lupus activity, immune‐mediated mechanisms, and immunosuppressive medications to the development and recurrence of IIH.

## 5 Conclusion

Recurrent idiopathic intracranial hypertension is a rare but potentially vision‐threatening neurological complication of systemic lupus erythematosus. Prompt recognition, exclusion of secondary causes, and timely treatment are essential to prevent permanent visual sequelae. Because both lupus activity and immunosuppressive therapy may contribute to intracranial hypertension, careful longitudinal assessment and multidisciplinary follow‐up are crucial. This case underscores the importance of considering medication‐related factors in patients with recurrent IIH and highlights the need for individualized therapeutic strategies.

## Author Contributions

**Leila Poorsaadat:** conceptualization, data curation, formal analysis, investigation, methodology, project administration, resources, supervision, validation, visualization, writing – review and editing. **Baharak Tasorian:** conceptualization, data curation, investigation, methodology, project administration, resources, supervision, validation, visualization, writing – review and editing. **Mohammad Sadegh Fakhari:** conceptualization, data curation, formal analysis, investigation, methodology, resources, writing – original draft. **Reza Mohammadi:** conceptualization, data curation, formal analysis, investigation, resources, writing – original draft.

## Funding

The authors have nothing to report.

## Ethics Statement

A written informed consent was obtained from the participant. Authors confirm that all methods were performed in accordance with institutional ethical standards and the Declaration of Helsinki.

## Consent

Written informed consent was obtained from the patient for publication of this case report and any accompanying images. A copy of the written consent is available for review by the Editor‐in‐Chief of this journal.

## Conflicts of Interest

The authors declare no conflicts of interest.

## Supporting information


**File S1:** Automated visual field examination performed after the first episode of idiopathic intracranial hypertension (October 2023). The examination demonstrates a visual field defect consistent with papilledema and elevated intracranial pressure.

**File S2:** Automated visual field examination performed during follow‐up after the first episode of idiopathic intracranial hypertension (March 2024). The examination demonstrates marked improvement of the previously documented visual field defect, consistent with clinical recovery following treatment.

**File S3:** Optical coherence tomography (OCT) findings obtained during the second episode of idiopathic intracranial hypertension (November 2024). OCT demonstrates bilateral optic nerve head edema, consistent with papilledema observed on funduscopic examination.

## Data Availability

All data are available from the corresponding author on reasonable request.
