# Supplementary material for: Recurrent Idiopathic Intracranial Hypertension in a Patient With Systemic Lupus Erythematosus: A Case Report
Source: Clin Case Rep. 2026 Aug 9;14(8):e73287. doi: 10.1002/ccr3.73287 (PMC13454553; doi:10.1002/ccr3.73287)
Supplement: Supplementary file 1 [file CCR3-14-e73287-s001.pdf]

Left eye (OS) / 03/09/2024 / 16:57:48

Seven-in-One

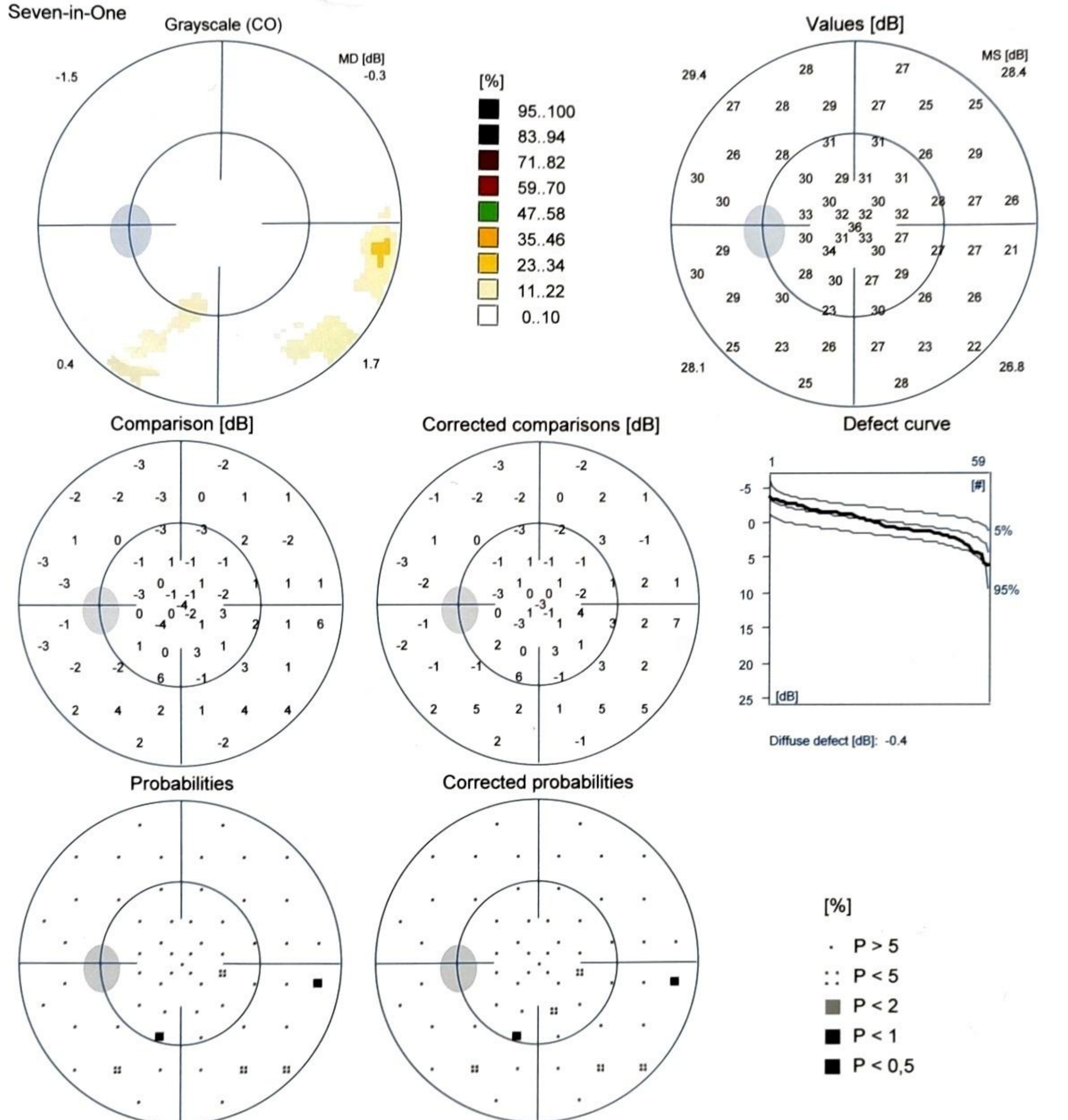

30°	
MS [dB]:	28.3
MD [< 2.0 dB]:	0.0
sLV [< 2.5 dB]:	2.3

**Programs:**	G Standard / White/White / 4000 / III Dynamic	**Questions / repetitions:**	142 / 1
**Parameters:**	31.4 / 4000 asb III 100 ms	**Duration:**	06:15
**Catch trials:**	1/7 (14%) *, 0/7 (0%)	**RF:**	7.1
**Refraction S/C/A:**	∅	**VA [m]:**	
**Pupil [mm]:**		**IOP [mmHg]:**	
**NV:**	T12 V2. 1		

Comment:

Right eye (OD) / 03/09/2024 / 16:40:38

Seven-in-One

Grayscale (CO)

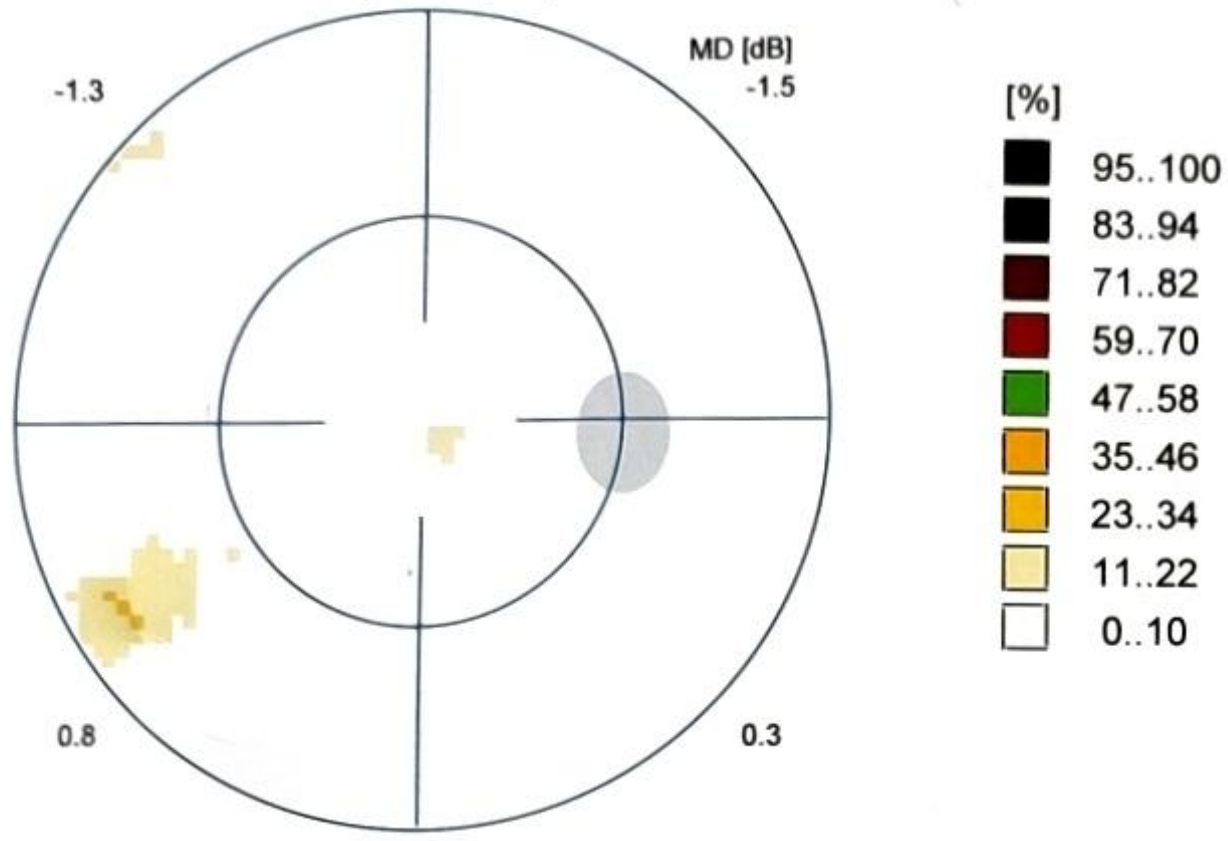

Values [dB]

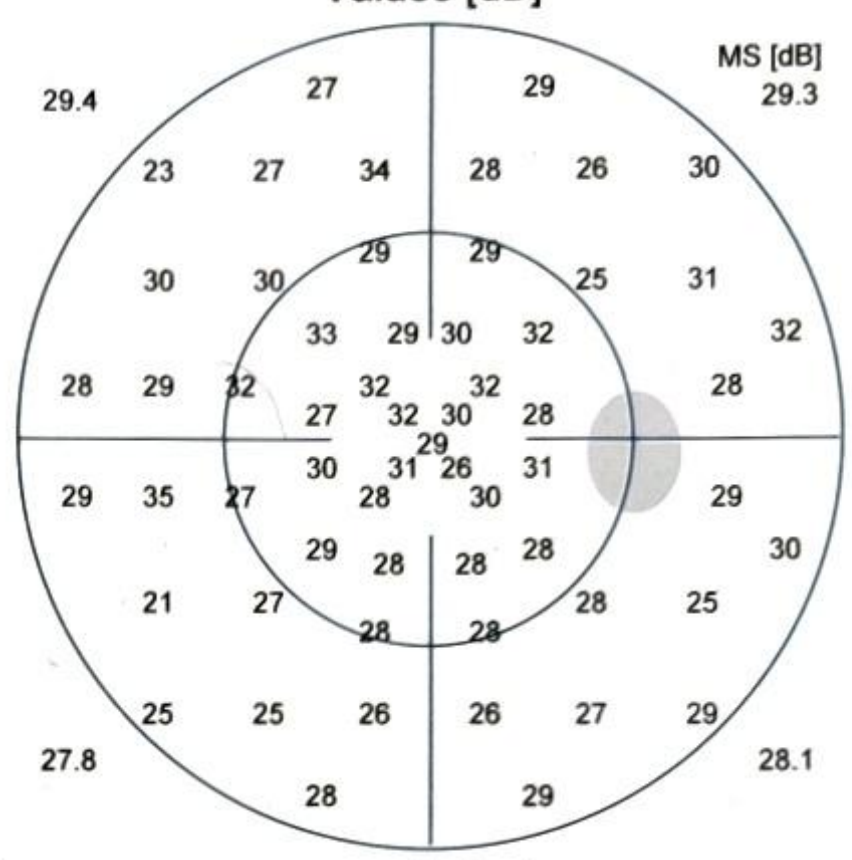

Comparison [dB]

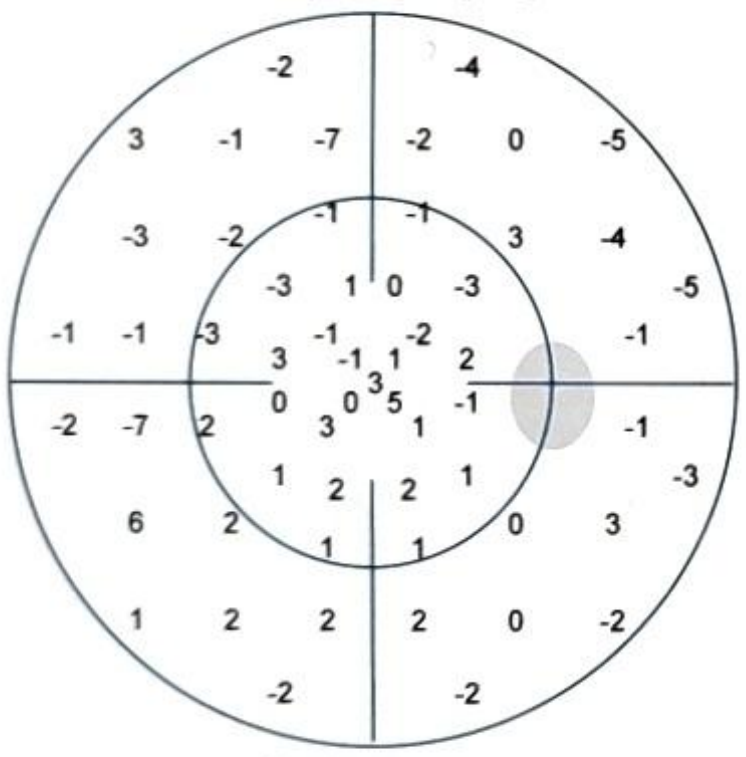

Corrected comparisons [dB]

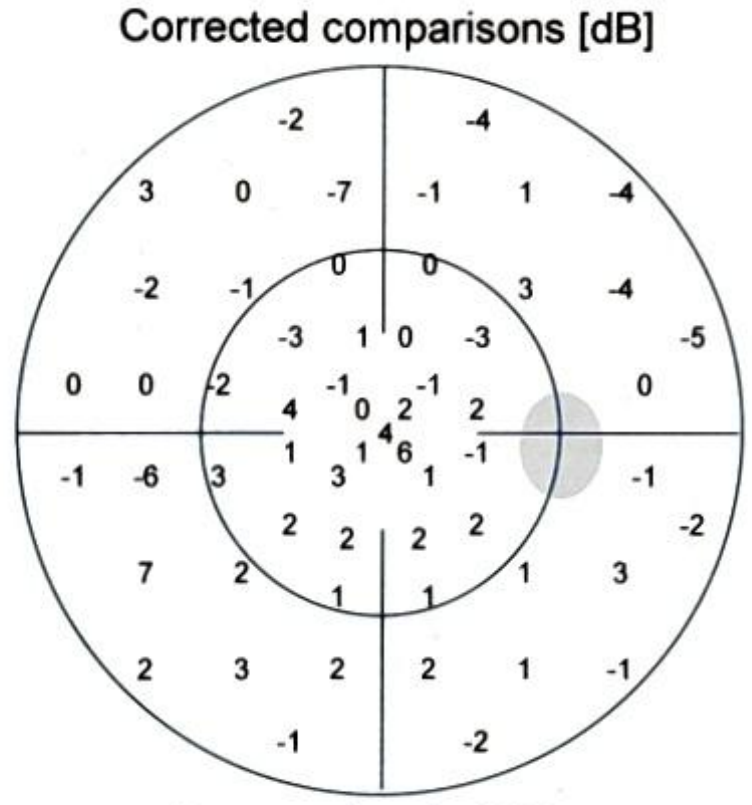

Defect curve

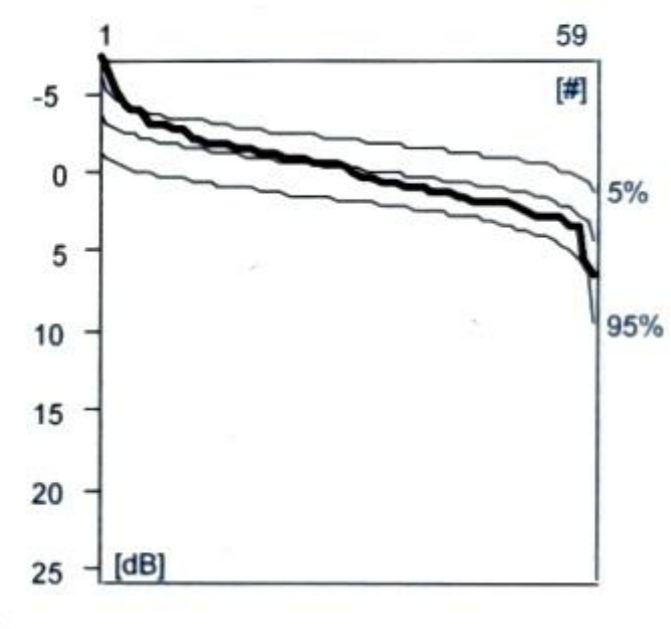

Diffuse defect [dB]: -0.6

Probabilities

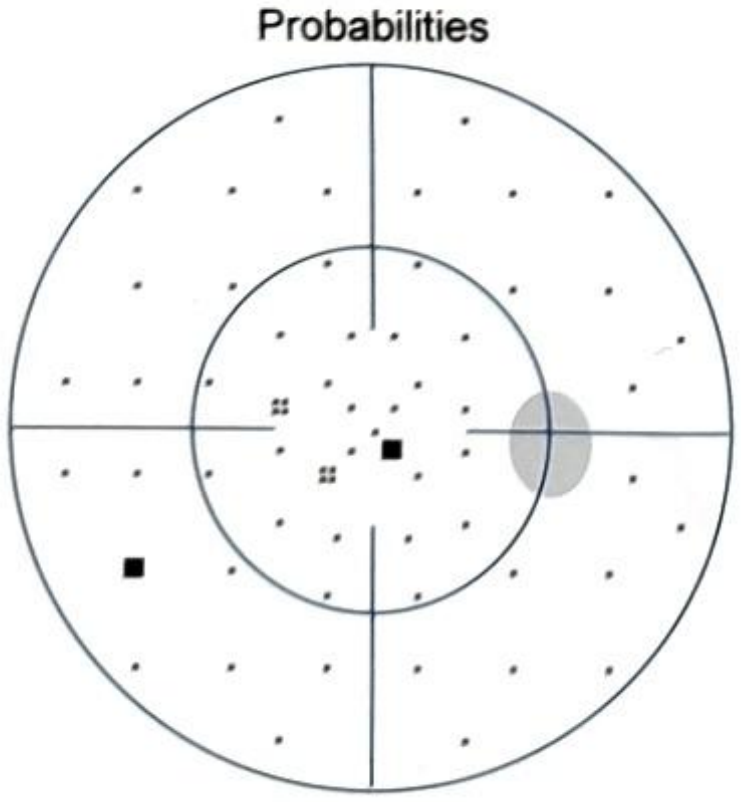

Corrected probabilities

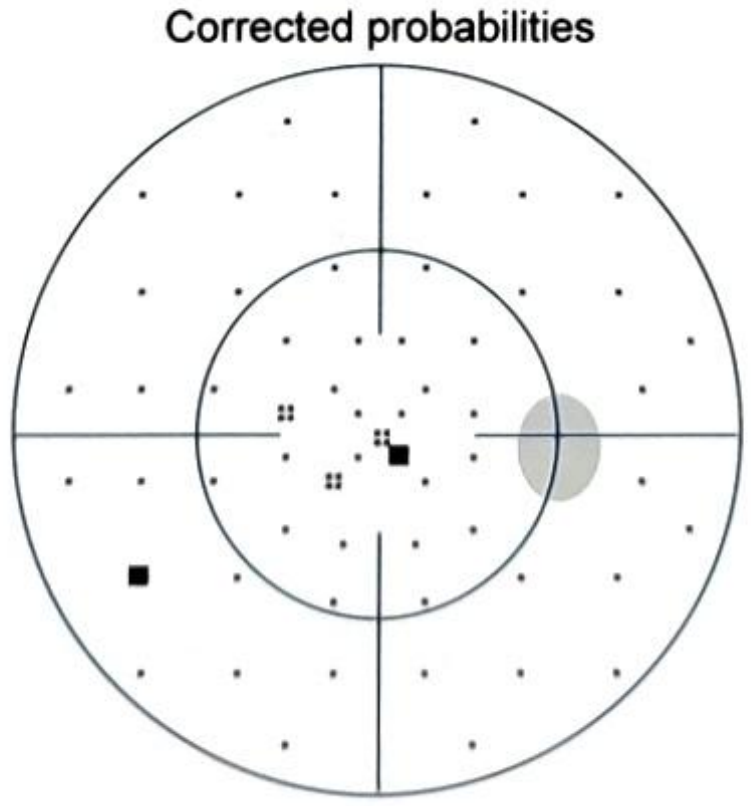

[%]

- P > 5
- P < 5
- P < 2
- P < 1
- P < 0,5

**Programs:**	G Standard / White/White / 4000 / III Dynamic
**Parameters:**	31.4 / 4000 asb III 100 ms
**Catch trials:**	1/8 (12%) +, 1/8 (12%)
**Refraction S/C/A:**	
**Pupil [mm]:**	
**NV:**	T12 V2 1

**Questions / repetitions:**	159 / 1
**Duration:**	05:58
**RF:**	12.5
**VA [m]:**	
**IOP [mmHg]:**	

30°	
**MS [dB]:**	**28.7**
**MD [< 2.0 dB]:**	**-0.3**
**sLV [< 2.5 dB]:**	**2.6**

Comment:
