# Supplementary material for: Recurrent Idiopathic Intracranial Hypertension in a Patient With Systemic Lupus Erythematosus: A Case Report
Source: Clin Case Rep. 2026 Aug 9;14(8):e73287. doi: 10.1002/ccr3.73287 (PMC13454553; doi:10.1002/ccr3.73287)
Supplement: Supplementary file 2 [file CCR3-14-e73287-s002.pdf]

Left eye (OS) / 10/02/2023 / 17:00:04

Seven-in-One

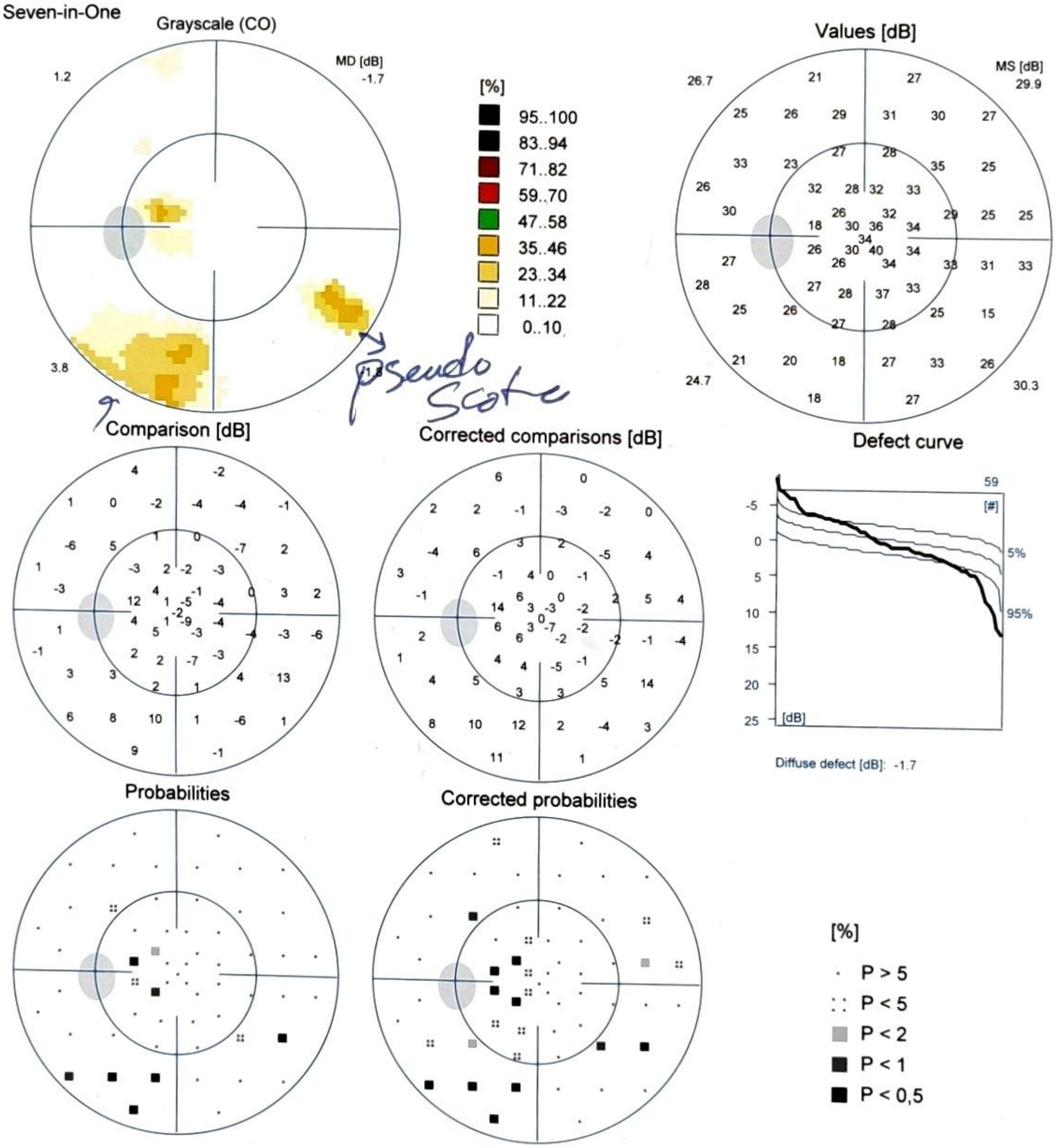

**Programs:**	G Standard / White/White / 4000 / III Dynamic	**Questions / repetitions:**	169 / 1
**Parameters:**	31.4 / 4000 asb III 100 ms	**Duration:**	10:12
**Catch trials:**	2/8 (25%) +, 0/9 (0%) -	**RF:**	11.7
**Refraction S/C/A:**	∅	**VA [m]:**	
**Pupil [mm]:**		**IOP [mmHg]:**	
**NV:**	T12 V2.1		

30°	
**MS [dB]:**	**28.1**
**MD [< 2.0 dB]:**	**0.3**
**sLV [< 2.5 dB]:**	**4.4**

Right eye (OD) / 10/02/2023 / 17:19:14

Seven-in-One

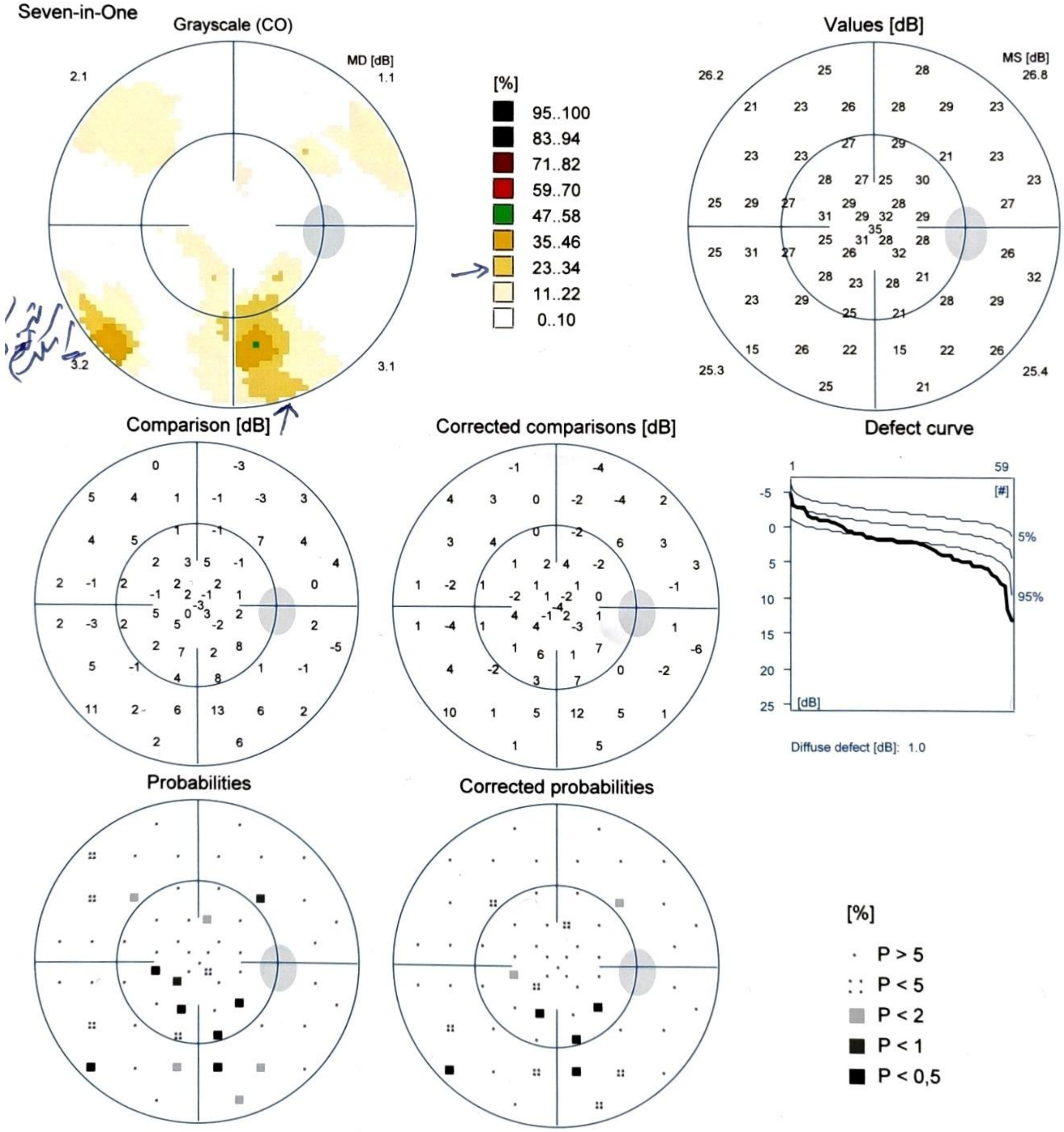

**Programs:**	G Standard / White/White / 4000 / III Dynamic	**Questions / repetitions:**	161 / 1
**Parameters:**	31.4 / 4000 asb III 100 ms	**Duration:**	08:56
**Catch trials:**	4/8 (50%) +, 0/8 (0%) -	**RF:**	25.0
**Refraction S/C/A:**		**VA [m]:**	
**Pupil [mm]:**		**IOP [mmHg]:**	
**NV:**	T12 V2.1		

30°	
**MS [dB]:**	**26.1**
**MD [< 2.0 dB]:**	**2.3**
**sLV [< 2.5 dB]:**	**3.4**
