# Supplementary material for: Recurrent Idiopathic Intracranial Hypertension in a Patient With Systemic Lupus Erythematosus: A Case Report
Source: Clin Case Rep. 2026 Aug 9;14(8):e73287. doi: 10.1002/ccr3.73287 (PMC13454553; doi:10.1002/ccr3.73287)
Supplement: Supplementary file 3 [file CCR3-14-e73287-s003.pdf]

TOPCON 3D Disc Report OU w/ Topography

ID:

Name:

Ethnicity: Caucasian
Gender: Female
Age: 28

Technician:
Fixation: Disc / Disc
Scan: 3D(6.0x6.0mm - 512x128)

Maestro2

OD(R)

Image Quality: 51 Analysis mode:Fine (2.0.7)
Capture Date: 2024/11/23

SuperPixel-200

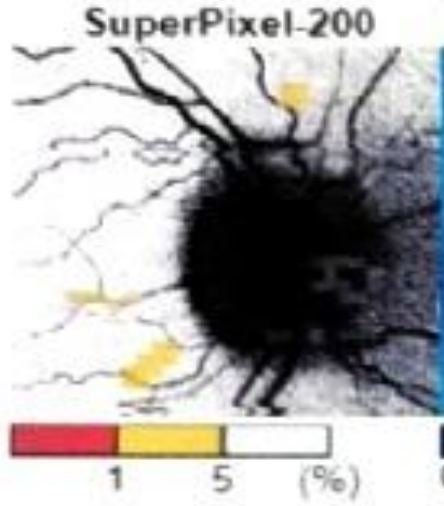

Thickness Map RNFL

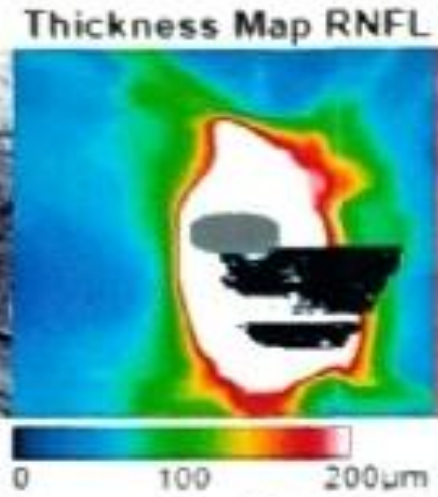

Color photo

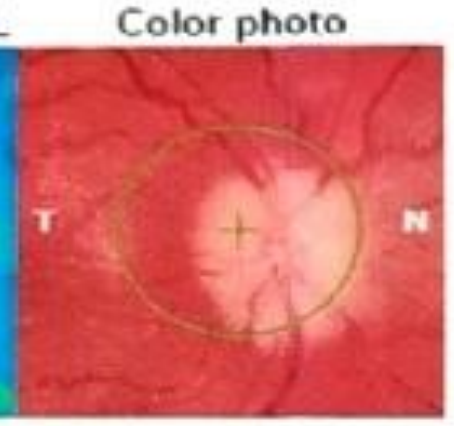

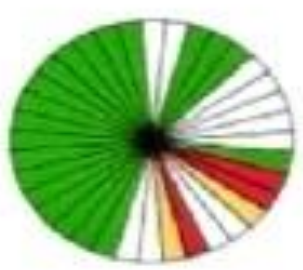

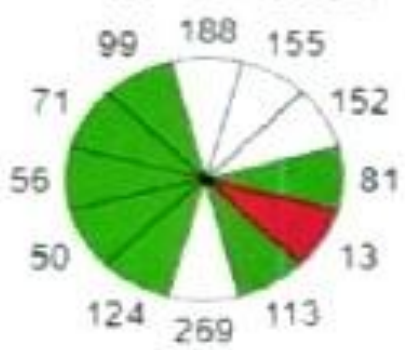

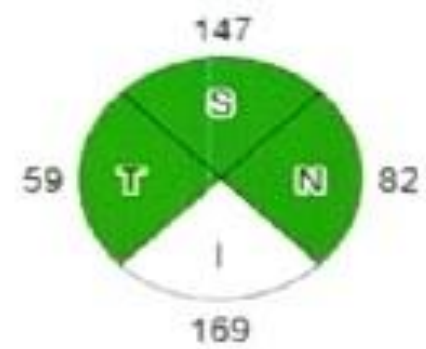

RNFL Circular Tomogram

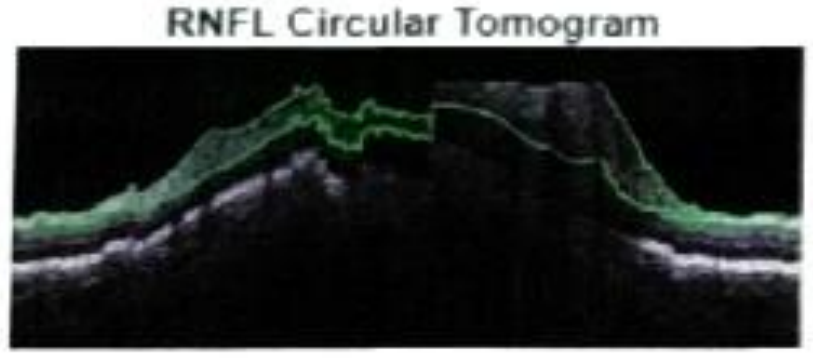

Reference plane view

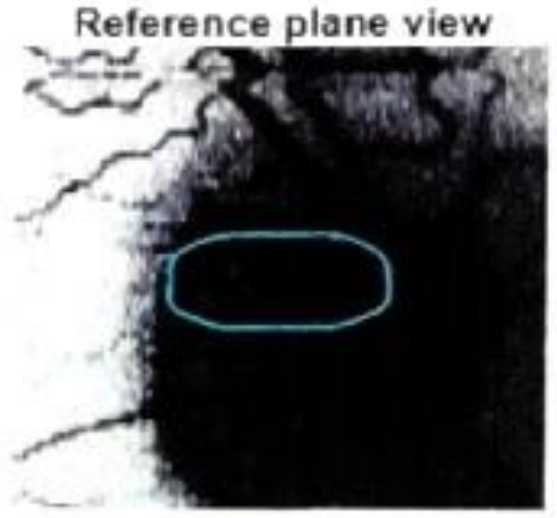

Red-free

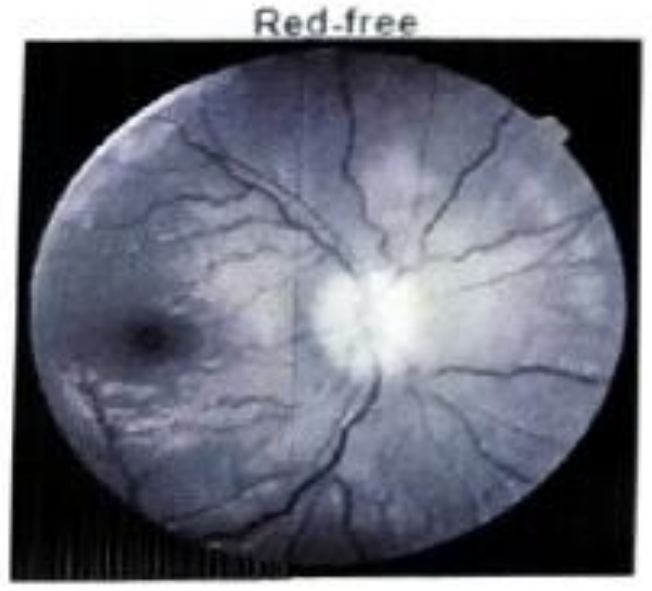

Horizontal Tomogram

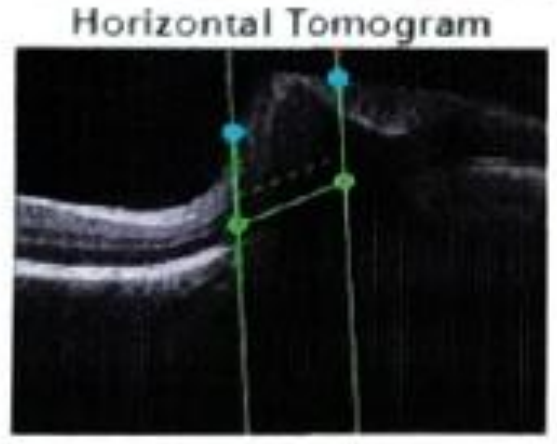

RNFL Circular Thickness Dia3.4mm

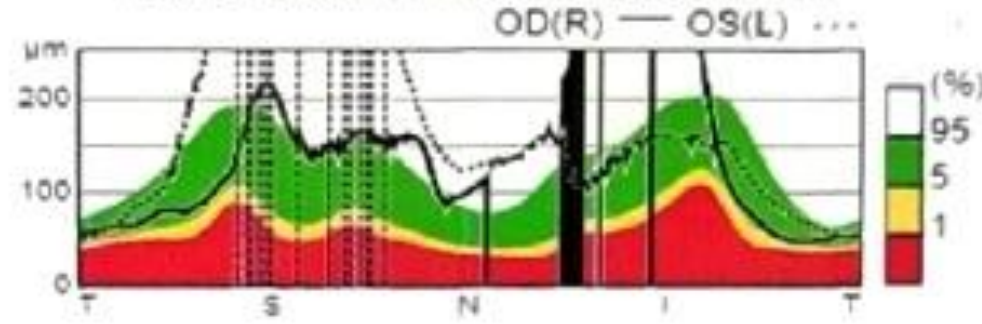

RNFL Symmetry 18%

Average thickness RNFL (µm)

OD(R)		OS(L)
114	Total Thickness	144
147	Superior	203
169	Inferior	145

Disc Topography

0.72	Rim Area	(mm^2)
0.72	Disc Area	(mm^2)
0.00	Linear CDR	
0.00	Vertical CDR	
0.00	Cup Volume	(mm^3)

Disc contour —— Cup contour ——

R/D Ratio

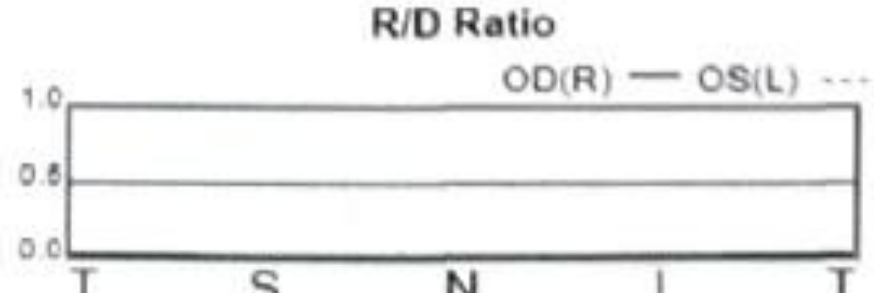

Disc parameters are determined at the reference plane height of (OD(R)120/OS(L)) microns from the RPE plane in this version.

OS(L)

Analysis mode:Fine (2.0.7) Image Quality: 46
Capture Date: 2024/11/23

Color photo

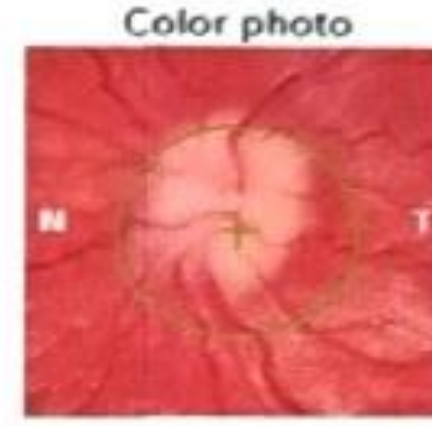

Thickness Map RNFL

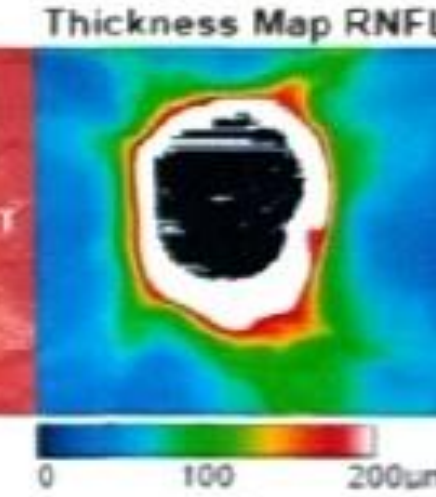

SuperPixel-200

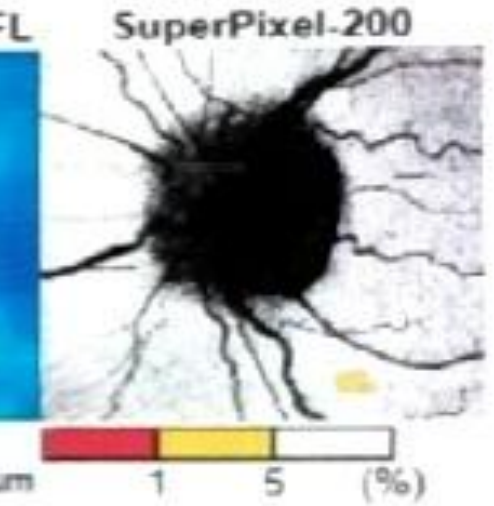

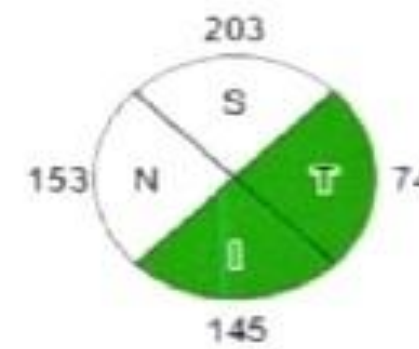

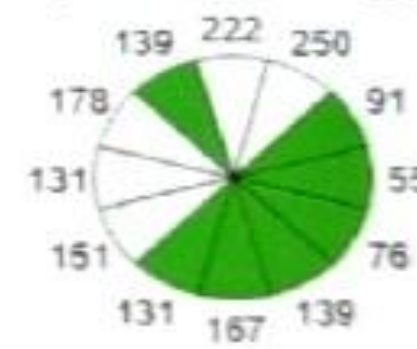

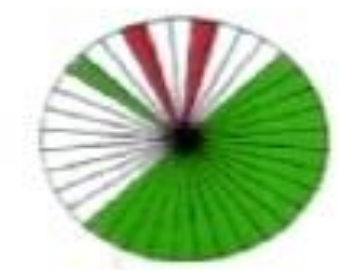

RNFL Circular Tomogram

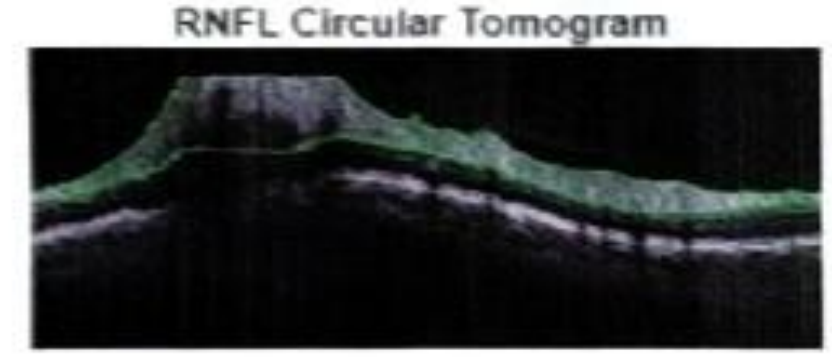

Red-free

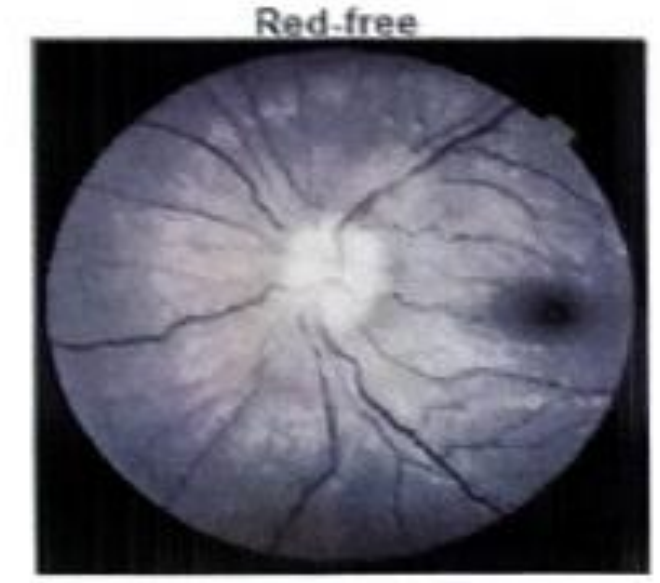

Comments:

Signature: Date:

Print Date: 2024/11/23 17:45:50
